# Preparation of TiO_2_ Nanocrystallite Powders Coated with 9 mol% ZnO for Cosmetic Applications in Sunscreens

**DOI:** 10.3390/ijms13021658

**Published:** 2012-02-03

**Authors:** Horng-Huey Ko, Hui-Ting Chen, Feng-Ling Yen, Wan-Chen Lu, Chih-Wei Kuo, Moo-Chin Wang

**Affiliations:** 1 Department of Fragrance and Cosmetic Science, Kaohsiung Medical University, 100 Shih-Chuan 1st Road, Kaohsiung 80708, Taiwan; E-Mails: hhko@kmu.edu.tw (H.-H.K.); htchen@kmu.edu.tw (H.-T.C.); flyen@kmu.edu.tw (F.-L.Y.); wclu@kmu.edu.tw (W.-C.L.); 2 Department of Resources Engineering, National Chen Kung University, 1 Ta-Hsueh Road, Tainan 70101, Taiwan; E-Mail: jeffreykuo@passivecomponent.com

**Keywords:** anatase, rutile, surface modification with 9 mol% TiO_2_, sunscreens cosmetic application, co-precipitation process

## Abstract

The preparation of TiO_2_ nanocrystallite powders coated with and without 9 mol% ZnO has been studied for cosmetic applications in sunscreens by a co-precipitation process using TiCl_4_ and Zn(NO_3_)_2_·6H_2_O as starting materials. XRD results show that the phases of anatase TiO_2_ and rutile TiO_2_ coexist for precursor powders without added ZnO (T-0Z) and calcined at 523 to 973 K for 2 h. When the T-0Z precursor powders are calcined at 1273 K for 2 h, only the rutile TiO_2_ appears. In addition, when the TiO_2_ precursor powders contain 9 mol% ZnO (T-9Z) are calcined at 873 to 973 K for 2 h, the crystallized samples are composed of the major phase of rutile TiO_2_ and the minor phases of anatase TiO_2_ and Zn_2_Ti_3_O_8_. The analyses of UV/VIS/NIR spectra reveal that the absorption of the T-9Z precursor powders after being calcined has a red-shift effect in the UV range with increasing calcination temperature. Therefore, the TiO_2_ nanocrystallite powders coated with 9 mol% ZnO can be used as the attenuate agent in the UV-A region for cosmetic applications in sunscreens.

## 1. Introduction

Fine particles of various metal oxides, such as titanium oxide (TiO_2_) and zinc oxide (ZnO) are extensively used as agents to attenuate (absorb and/or scatter) ultraviolet (UV) radiation, and have many describable characteristics, such as a long history of topical use, broad spectrum absorption, high photostability and low irritancy [1]. Nohynek *et al.* [2] have reported that modern sunscreens contain insoluble TiO_2_ or ZnO nanoparticles due to those are colorless and reflect/scatter UV light more efficiently than larger particles. The crystal structure of TiO_2_ has three different polymorphic forms: brookite (orthorhombic), anatase (tetragonal) and rutile (tetragonal). The UV-attenuating effect of TiO_2_ is dependent on the crystal structure, surface area, size distribution, porosity, surface hydroxyl density, *etc.*, as reported by Lee *et al.* [3]. However, TiO_2_ powders attenuate UV radiation more effectively only in the UVB region.

To increase the transparency and attenuate the UV radiation more effectively with a lower content of metal oxide particles, nano-sized primary single particles must be dispersed homogeneously into the medium [4]. In addition, the surface passivation of metal oxide nanoparticles with a layer of inorganic, organic, or bioactive materials to form the core-shell nanoparticles has attracted considerable attention [5]. A surface coating can be added to nanosized TiO_2_ to enhance its UV absorption by the diffraction mechanism of different light. The TiO_2_ surface coating with silica enhances UV-B absorption, but not UV-A absorption, as reported by Jaroenworaluck *et al.* [6]. Liao *et al.* [7] pointed out that the absorbance of amorphous TiO_2_-coated ZnO nanoparticles at 375 nm gradually decreased with an increase in the molar ratio of Ti/Zn and time for the TiO_2_ coating, and that the emission intensity of ZnO cores could be significantly enhanced by the amorphous TiO_2_ shell. However, the studies of the TiO_2_ nanocrystallite powders coated with ZnO for sunscreens cosmetic applications have not been discussed in detail.

In order to promote the absorption of TiO_2_ nanocrystallite powders to UV-A region, the surface of TiO_2_ nanocrystallite powders must be modified. In the present work, an effort on the surface modification of TiO_2_ nanocrystallite powders coated with 9 mol% ZnO for cosmetic applications in sunscreens was studied using X-ray diffraction (XRD), transmission electron microscopy (TEM), selected area electron diffraction (SAED), and UV-vis absorption spectra. The aims of this paper are: (i) to study the phase transformation of TiO_2_ precursor powders with and without 9 mol% ZnO; (ii) to observe the microstructure of TiO_2_ nanocrystallite powders with 9 mol% ZnO; and (iii) to evaluate the UV absorption at 200–700 nm.

## 2. Results and Discussion

**Figure 1:**
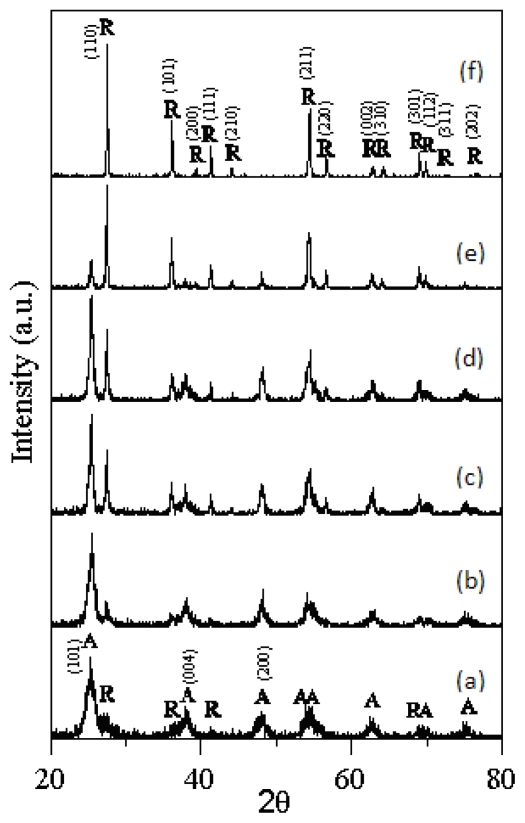
X-ray diffraction (XRD) patterns of the T-0Z freeze dried precursor powders are calcined at various temperatures for 2 h: (**a**) 523 K; (**b**) 673 K; (**c**) 773 K; (**d**) 873 K; (**e**) 973 K and (**f**) 1273 K (A: anatase, R: rutile).

Figure 1 shows the XRD patterns of the T-0Z freeze dried precursor powders are calcined at various temperatures for 2 h. Figure 1a shows the T-0Z precursor powders are calcined at 523K for 2 h, and reveals that the crystalline phases of the powders were composed of the anatase and rutile TiO_2_, but the crystallinity of rutile TiO_2_ was very poor. The crystallinity of rutile increased while the anatase decreased as the calcination temperature rose from 673 to 973 K (Figure 1b–d). When calcined at 1273 K for 2 h, XRD result (Figure 1f) shows that only rutile TiO_2_ appeared.

In the present study, the rutile TiO_2_ in T-0Z freeze dried precursor powders began to form at 523 K, and was the dominant phase when calcined at 973 K, and saw further increases as the calcination temperature rose. The anatase to rutile TiO_2_ transformation is affected by crystallite size, dopant type and concentration, as well as the titanium oxide precursor in solution chemical synthesis, as reported by Zhang and Banifield [8]. In fact, the phase transformation of anatase to rutile TiO_2_ is nucleated at anatase {112} twin boundaries, and the rutile nucleation involves the displacement of only half the titanium cations in the twin slab [9]. When the transformation is occurs continuously, slabs of anatase octahedral are destabilized, which results in a rapid progression of anatase into rutile TiO_2_.

**Figure 2:**
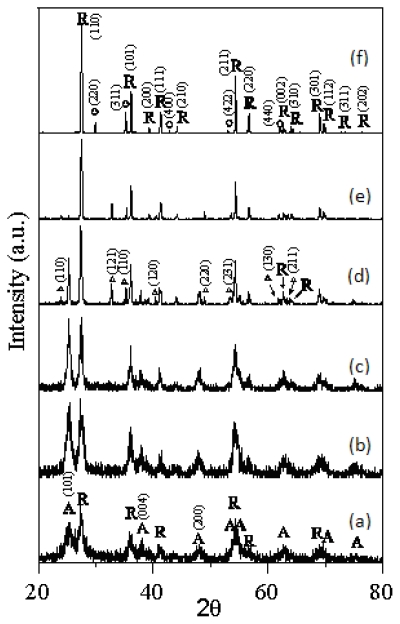
XRD patterns of the T-9Z freeze dried precursor powders are calcined at various temperatures for 2 h: (**a**) 523 K; (**b**) 673 K; (**c**) 773 K; (**d**) 873 K; (**e**) 973 K and (**f**) 1273 K (A: anatase; R: rutile; Δ: Zn_2_Ti_3_O_8_; ○: Zn_2_TiO_4_).

Figure 2 shows the XRD patterns of the T-9Z freeze dried precursor powders are calcined at various temperatures for 2 h. Figure 2a shows the XRD pattern of the T-9Z freeze dried precursor powders are calcined at 523 K for 2 h, and reveals that the crystallites are the coexisting phases of anatase and rutile TiO_2_. It can also be seen in Figure 2b–f that the crystallinity of the rutile phase improved with the calcination temperature increase. However, the crystallinity of the anatase phase decreased with the rising calcination temperature. When calcined at 1273 K for 2 h (Figure 2f), the anatase TiO_2_ phase disappeared, but the rutile TiO_2_ still appeared. In addition, when the T-9Z freeze dried precursor powders are calcined at 873 K for 2 h, the minor phase of Zn_2_Ti_3_O_8_ first appeared (Figure 2d). Moreover, the Zn_2_Ti_3_O_8_ transformed to Zn_2_TiO_4_ when the T-9Z freeze dried precursor powders are calcined at 1273 K for 2 h (Figure 2f).

In addition, although zinc oxide is not identified in the XRD patterns for the present study, Figure 2e indicates that the phases of Zn_2_Ti_3_O_8_ and Zn_2_TiO_4_ were formed when T-9Z freeze dried precursor powders were calcinaed between 773 K and 1273 K for 2 h, respectively. The ionic radius of Ti^4+^ and Zn^2+^ are 0.68 Å and 0.74 Å, respectively. Since these values are almost equal, it can be inferred that the zinc ions did not insert into the structure of titanium, and instead were located at interstices or absorbed on the surface of TiO_2_, thus forming the zinc-titanium solid solution [10].

The average crystallite size of the T-0Z and T-9Z freeze dried precursor powders calcination at various temperatures for 2 h is determined by Scherrer’s formula [11]:

(1) $$\text{D}=\frac{0.9\lambda }{\beta cos\theta }$$

where D is the average crystallite size of the anatase and rutile TiO_2_, λ = 0.154 nm is the X-ray wavelength of CuKα, β is the full width of the peak measured at half maximum intensity and θ is the Bragg’s angle of the peak.

The average crystallite size of the anatase and rutile phases TiO_2_ after it has been calcined at various temperatures for 2 h is obtained and listed in Table 1. It can be seen that the average crystallites of anatase and rutile TiO_2_ increase with calcination temperature increased but all sizes are smaller than 100 nm. These results reveal all crystallite sizes of anatase and rutile TiO_2_ in the nano-scale. Moreover, the crystallite size of rutile TiO_2_ in T-0Z is smaller than that in T-9Z when the freeze dried precursor powders are calcined at 1273 K for 2 h.

It is well known that the polymorphic transformation of ceramic materials generally depends on the nature of the dopant, amount of the dopant and the processing route. The additions of Cr_2_O_3_ [12], SiO_2_ [13], and CeO_2_ [14,15] have been found to retard the anatase to rutile TiO_2_ transformation. Furthermore, the additions of Fe_2_O_3_ [16] and AlCl_3_ [17] have been shown to enhance the anatase to rutile TiO_2_ transformation. In the present study, for the samples of T-0Z and T-9Z, the diffraction peaks show the anatase and rutile phases of TiO_2_ were simultaneously present from 523 to 973 K, but when calcined at 973 K for 2 h, the (110) intensity of rutile TiO_2_ was greater than the (101) intensity of anatase TiO_2_ for the T-0Z sample. In addition, when the T-9Z sample are calcined of 973 K for 2 h, the (101) intensity of anatase TiO_2_ was only residual, and thus insignificant. Comparing Figures 1a and 2a, it can be observed that the (110) intensity of anatase is greater than the (110) intensity of rutile TiO_2_ in Figure 1a, but the results are different in Figure 2a. These results prove that doping ZnO into TiO_2_ can enhance the anatase to rutile TiO_2_ transformation and shift the transformation to a lower temperature. This phenomenon occurs because the zinc oxide leading to the surface nucleation occurred on this polymorth [18].

The influence of the dopant ZnO on the structure of the T-9Z samples can be explained based on the changes caused by the dopant on the TiO_2_ surface [14]. Because the ionic radius of Zn^2+^ (0.74 Å) is greater than that of Ti^4+^ (0.68 Å), but smaller than that of oxygen (1.32 Å), the zinc ions were not introduced into the structure of titanium oxide matrix [10]. Therefore, the same deformation of the lattice structure and deformation energy produced by the zinc ions did not occur, and the zinc ions absorbed on the surface of titanium oxide created the nucleation sites and enhanced an unstable anatase phase transition to rutile. The anatase to rutile TiO_2_ transformation was nearly complete at 973 K, as the surface nucleation is favored for the dopant.

Moreover, although the concentration was only 9.0 mol% ZnO, the segregation of dopant on the surface of titanium oxide matrix promoted the Zn_2_Ti_3_O_8_ formation at 873 K in the ZnO-TiO_2_ system. Chang *et al.* [19] synthesized zinc titanate nanocrystal powder using a sol-gel process, and noted that the phase of the low temperature form of ZnTiO_3_ (*i.e.*, Zn_2_Ti_3_O_8_) first formed at 773 K, but had poor crystallinity. At 873 K, the crystalline phase of ZnTiO_3_ was identified, but traces of Zn_2_TiO_4_ and rutile also appeared. Wang *et al.* [20] using hydrothermal process prepared the Zn_2_Ti_3_O_8_ powders for cosmetic applications have reported that the zinc titanium powders was obtained from the TiCl_4_, Zn(NO_3_)_2_·6H_2_O and NH_4_OH solutions put in a thermostatic autoclave at 423 K for 1 h. When the zinc titanate powders are calcined at 873 K for 1 h, the Zn_2_Ti_3_O_8_, ZnO and anatase TiO_2_ coexisted. Dulin and Rase [10] have pointed out that when the ZnO content is less than 50 mol% and the temperature below 1218 K, only the phases of Zn_2_Ti_3_O_8_ and rutile TiO_2_ are present. In addition, the Zn_2_TiO_4_ and rutile TiO_2_ appeared when the temperature was between 1218 and 1691 K. The results of the present study are in agreement with those of Dulin and Rase [10], and Chang *et al.* [19].

**Figure 3:**
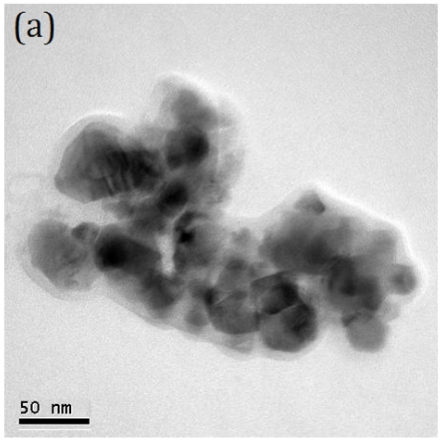

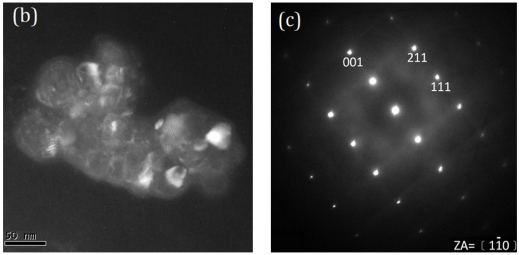
Transmission electron microscopy (TEM) microstructure and selected area electron diffraction (SAED) pattern of the T-0Z freeze precursor powders are calcined at 1273 K for 2 h: (**a**) bright field (BF) image; (**b**) dark field (DF) image and (**c**) SAED pattern. The SAED pattern corresponding to the rutile TiO_2_ with ZA = [11̄0].

Figure 3 shows the bright field (BF) and dark field (DF) images, and selected area electron diffraction (SAED) pattern of T-0Z freeze dried precursor powders are calcined at 1273 K for 2 h. Figure 3a shows the BF image of the irregular crystallites morphology with a size of about 60 nm. In addition, Figure 3b shows the DF image of the Figure 3a. Figure 3c reveals the SAED pattern corresponding to the rutile TiO_2_ with zone axes (ZA) of [11̄0]. The SAED pattern also provides the evidence of the presence of the rutile TiO_2_ in T-0Z powders when calcined at 1273 K for 2 h. Moreover, the crystallite sizes in Figure 3a also correspond to the results of Table 1.

**Figure 4:**
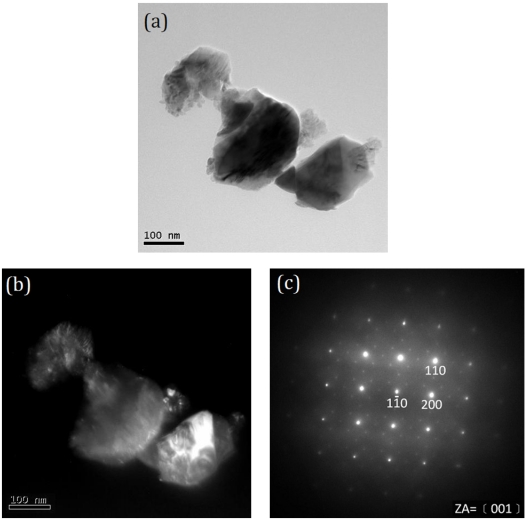
TEM microstructure and SAED pattern of the T-9Z freeze dried precursor powders calcined at 1273 K for 2 h: (**a**) BF image; (**b**) DF image and (**c**) SAED pattern. The SAED pattern corresponding to the rutile TiO_2_ with ZA = [001].

When the T-9Z freeze dried precursor powders are calcined at 1273 K for 2 h, the TEM microstructure and SAED pattern are shown in Figure 4. Figure 4a,b shows the BF and DF images, respectively. The crystallite sizes are observed to be about 80 nm. Figure 4c shows the SAED pattern of Figure 4b. The SAED pattern corresponds to the rutile TiO_2_ with ZA = [001].

**Figure 5:**
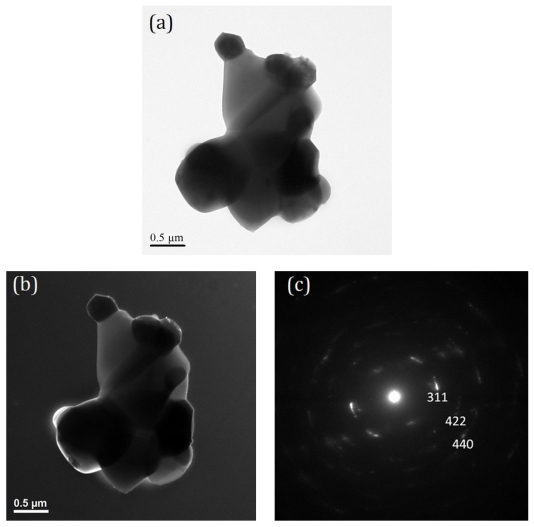
TEM microstructure and SAED pattern of the T-9Z freeze dried precursor powders are calcined at 1273 K for 2 h: (**a**) BF image; (**b**) DF image; and (**c**) SAED pattern of fringe on (**b**). The SAED pattern corresponding to the Zn_2_TiO_4_.

Figure 5a shows the BF image of the aggregate rutile crystallites in the irregular larger particle. Furthermore, the Figure 5b shows the fringe of the aggregate rutile TiO_2_. Figure 5c shows the SAED pattern of the fringe in Figure 5b. The SAED pattern corresponds to the Zn_2_TiO_4_. The results from Figures 4c and 5c correspond to those of Figure 2f.

**Figure 6:**
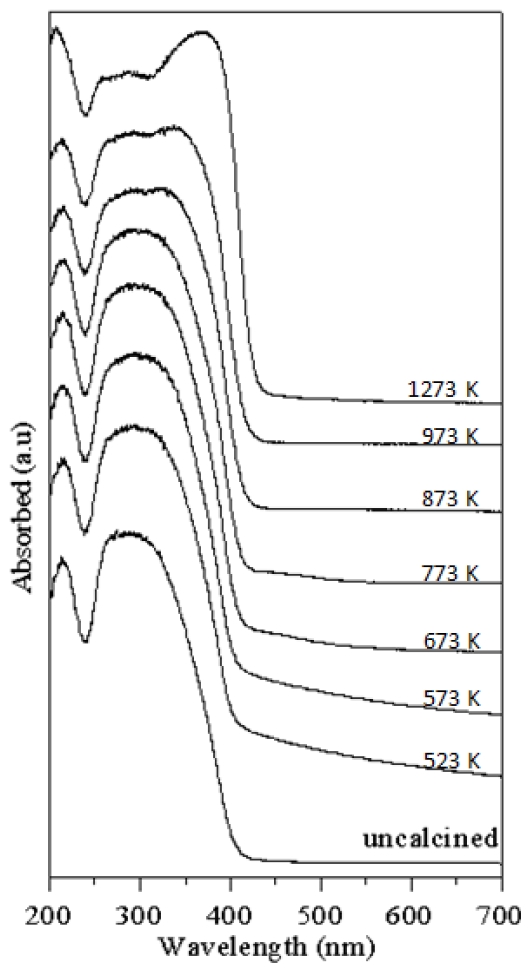
Relation of the absorbed and wavelength range between 200 and 700 nm of the T-9Z freeze dried precursor powders calcined at various temperatures for 2 h.

The relation of absorption and wavelength range between 200 and 700 nm for T-9Z freeze dried precursor powders calcined at various temperatures for 2 h are shown in Figure 6. It is found that the absorption of T-9Z powders in the UV range had a red-shift effect as the calciantion temperature increased. This is because the quantity of photons reaching the core of a particle depends of the size and the optical properties of the TiO_2_ crystals. Maris *et al.* [21] also found that the penetration of light into a particle is influenced by the superficial morphology of the particles. Particles formed from larger TiO_2_ crystals have a smoother surface than those made from small crystals. On a smooth surface, the incident photons are scattered and mostly lost by reflection. In contrast, a rough surface allows a greater number of scattered photons to penetrate into the particle [15]. Moreover, the red-shift effect of the TiO_2_ nanocrystallite powders with surface modified by 9 mol% ZnO and calcined at 1273 K for 2 h indicates that they can be used as an UV-A attenuating agents for cosmetic applications in sunscreens.

Most of the UV absorbers used in sunscreens are oil-soluble or even oil-miscible and consequently are incorporated into the oil phase of sunscreen emulsions [22]. Moreover, Herzog *et al.* [22] also pointed out that the UV-attenating efficiency increased with decreasing particle size up to a maximum particle size of 80 nm. With smaller particles, the extinction started to decrease again. Moreover, for the inorganic TiO_2_ used as absorber at a particle size of 100 nm, the scattering that contributes to about 50% to the overall extinction in the range of the extinction maximum of the spectrum has been reported by Robb *et al.* [23]. On the other hand, Popov *et al.* [24] pointed out that the TiO_2_ fine particles are embedded with sunscreens into the skin to effectively attenuate UV-B radiation. Moreover, TiO_2_ particles with a size of 62 nm are found to be the most effective in protecting skin against UV-B radiation. As mentioned above, the results of the present study show a red-shift effect in the UV range. Therefore, the TiO_2_ nanocrystallite powders with surface modified by 9 mol% ZnO and calcined at 1273 K for 2 h can be used as an UV-A light attenuating agent for cosmetic applications in sunscreens.

## 3. Experimental Procedure

### 3.1. Sample Preparation

The starting materials were reagent-grade TiCl_4_ solution (purity ≥ 98.0%, supplied by Fluka, France), Zn(NO_3_)_2_·6H_2_O (purity ≥ 98%, supplied by Alfa Aersor, USA), 25 vol% ammonia solution (NH_4_OH, supplied by Riedel-de Haën, Germany) and polyethylene glycol (PEG, supplied by Nippon Shiyaku Kogyo K.K., Japan). A TiCl_4_ solution of 0.5 M was prepared from 1 mol TiCl_4_ solution dissolved in 2 L deionized water. The TiCl_4_ aqueous solution was then supplemented with 1 wt% of PEG as a dispersant. The mixed solution was stirred and heated to 333 K for 6 h (denoted as solution I). NH_4_OH was then added to solution I until pH = 9. The mixed solution was then stirred at room temperature for 24 h to obtain white precipitates (denoted as T-0Z).

An aqueous solution of 9 mol% ZnO was prepared from the Zn(NO_3_)_2_·6H_2_O dissolved in deionized water and stirred at 273 K for 2 h (denoted as solution II). Solution II was then added slowly at a rate of 0.05 cm^3^·min^−1^ with vigorous agitation in solution I at room temperature. At the end of titration, a syringe was used to minimize the size of falling drops and reduce the local reaction effect. During the whole process, the pH value was kept at 9 by adjusting the amount of NH4OH. The mixed solution was then stirred at room temperature for 24 h to obtain white precipitates (denoted as T-9Z).

After precipitation, the precipitates were washed thoroughly two times with a large amount of ethanol (purity ≥ 99.85%, supplied by J.T.Baker, USA) to remove NH_4_NO_3_ [25]. Subsequently, the precipitates were freeze dried at 218 K in a vacuum.

### 3.2. Sample Characterization

The crystalline phase was identified using an X-ray diffractometer (XRD, model Rad IIA, Rigaku Co., Tokyo, Japan) with Cu Kα radiation and a Ni filter, operated at 30 kV, 20 mA and a scanning rate (2θ) of 0.25°·min^−1^. The microstructure of the powders before and after calcination was observed by transmission electron microscopy (TEM, model HF-2000, Hitachi Ltd., Tokyo, Japan), operating at 200 kV. The selected area electron diffraction (SAED) examination was conducted on the calcined samples. The UV-shielding was measured with a UV-vis spectrometer (Optometrics, SPF-290, Ayer, MA, USA).

## 4. Conclusions

1. The rutile TiO_2_ began to form at 523 K of T-0Z freeze dried precursor powders, and was the dominant phase when calcined at 973 K for 2 h.
2. The anatase and rutile TiO_2_ phases coexist in T-9Z powders when the calcination temperature is below 973 K. When calcined at 1273 K for 2 h, the anatase phase disappears. In addition, the Zn_2_Ti_3_O_8_ first forms when T-9Z freeze dried precursor powders are calcined at 973 K for 2 h. When the T-9Z precursor powders are calcined at 1273 K for 2 h, the Zn_2_TiO_4_ forms and the Zn_2_Ti_3_O_8_ disappears.
3. The average crystallite sizes of anatase and rutile TiO_2_ increase with increasing the calcination temperature, but all average crystallite sizes of anatase and rutile TiO_2_ are smaller than 100 nm for T-0Z and T-9Z freeze dried precursor powders as calcined between 523 and 1275 K for 2 h. In addition, the crystallite size of rutile TiO_2_ in T-0Z is smaller than that in T-9Z when the freeze dried precursor powders are calcined at 1273 K for 2 h.
4. The absorption of T-9Z powders in the UV range has a red-shift effect as the calcination temperature increases. This result shows that TiO_2_ nanocrystallite powders added with 9 mol% ZnO and calcined at 1273 K for 2 h can be used as an UV-A attenuating agent for cosmetic applications in sunscreens.

## Figures and Tables

**Table 1:** The average crystallite size of anatase and rutile TiO_2_ when T-0Z and T-9Z freeze dried precursor powders are calcined at various temperatures for 2 h.

Calcination Temperature (K)	Crystallite Size T-0Z (nm)		Crystallite Size T-9Z (nm)	

	Anatase	Rutile	Anatase	Rutile
523	6.5 ± 0.2	-	5.0 ± 0.2	5.7 ± 0.2
673	9.8 ± 0.3	13.2 ± 0.2	8.6 ± 0.2	10.0 ± 0.2
773	15.5 ± 0.3	21.0 ± 0.4	12.9 ± 0.2	15.7 ± 0.2
873	16.5 ± 0.3	28.9 ± 0.4	31.4 ± 0.4	34.0 ± 0.4
973	20.4 ± 0.4	37.4 ± 0.5	-	48.6 ± 0.4
1273	-	57.7 ± 0.6	-	78.8 ± 0.6

“-” denotes the phase disappeared.
